# Gene-environment interaction in expertise acquisition: Practice effects on musical expertise vary by polygenic scores for cognitive performance

**DOI:** 10.1016/j.heliyon.2024.e34264

**Published:** 2024-07-06

**Authors:** Laura W. Wesseldijk, Miriam A. Mosing, Fredrik Ullén

**Affiliations:** a Department of Cognitive Neuropsychology, Max Planck Institute for Empirical Aesthetics, Frankfurt am Main, Germany; b Department of Psychiatry, Amsterdam UMC, University of Amsterdam, the Netherlands; c Department of Neuroscience, Karolinska Institutet, Sweden; d Melbourne School of Psychological Sciences, Faculty of Medicine, Dentistry, and Health Sciences, University of Melbourne, Australia; e Department of Medical Epidemiology and Biostatistics, Karolinska Institutet, Sweden

## Abstract

Expert performance is associated with practice, partly because of causal effects of practice on skill (i.e., learning). However, the practice-expertise association is also influenced by a complex interplay between genes and environment including partly overlapping genetic influences. The importance of cognitive ability in the practice-expertise association is less well understood. Therefore, we first examined whether genetic predisposition for cognitive performance, operationalized as a polygenic score, is associated with music practice and expertise. Next, we tested whether there is evidence for gene × environment interaction, i.e., whether effects of practice on expertise differ depending on an individual's genetic predisposition for cognitive performance. Polygenic scores for cognitive performance (PGScp) and multi-trait cognitive performances, including educational attainment and mathematical performances (PGScps) were calculated for approximately 3800 genotyped Swedish individuals with information available on their cumulative amount of music practice, musical achievement, and musical auditory discrimination. We found that higher PGScp and PGScps were associated with higher levels of achievement, musical auditory discrimination, and more practice, although the association with practice weakened when controlling for education. Music practice was linked to both expertise outcomes, and the effect sizes of these associations varied depending on an individual's PGScp and PGScps (with the exception of PGScp for musical auditory discrimination). These results suggest genetic pleiotropy between cognitive performance and musical expertise. Additionally, they reveal the presence of G × E interaction in skill acquisition, as effects of practice on musical expertise are stronger for individuals with a higher genetic predisposition for cognitive performance.

## 1 Introduction

Historically, research in expertise had a strong focus on how skilled performance is shaped by long-term practice. In influential early work, Ericsson and co-workers focused specifically on the impact of what they called deliberate practice, i.e., explicit, effortful, goal-directed activities designed to improve performance, and which may include guidance from a teacher [1,2]. While this research programme has provided ample evidence that goal-directed practice can lead to dramatic performance improvements, later work has demonstrated that the acquisition of expertise is a complex process which depends on numerous variables, apart from practice, such as physical traits or personality [3]. Furthermore, the benefits of practice vary from person to person, with some people learning much from a few hours of practice, and others making slower progress [4,5]. As a consequence, experts at comparable levels of skill and achievement show large variation on measures of cumulative practice [6,7].

One reason for this variation is that individual differences in expert performance, just as in essentially all human traits, are the result of environmental and genetic influences and their interplay. Our knowledge of gene-environment interplay underlying expertise is mainly based on twin research in the music domain. One of the more important findings from these twin studies is that not only musical expertise, but also music practice in itself, which traditionally has been regarded as an environmental influence, is influenced by genetic factors, with an estimated heritability of 41–69 % [8]. Additionally, the relation between music practice and at least some aspects of musical expertise, such as the ability to discriminate between different melodies, pitches or rhythms, is strongly influenced by familial (genetic and rearing environmental) factors [8]. Another notable finding is that the relationship between music practice and general cognitive ability – traditionally interpreted as far transfer effects on intelligence – could in fact be entirely explained by shared genetic factors, so-called genetic pleiotropy [9]. Similarly, pleiotropy plays a major role for the association between musical auditory discrimination and cognitive ability [10].

General cognitive ability, sometimes referred to as general intelligence, is the capacity to ‘reason, plan, solve problems, think abstractly, comprehend complex ideas, learn quickly, and learn from experience’ [11] and is relatively strongly influenced by genetic factors with heritability estimates based on twin studies ranging between 40 and 70 % [12,13]. Previous research suggests that the relationship between cognitive ability, music practice, and musical expertise is intricate, with higher cognitive ability being associated with larger practice effects. In other words, on the phenotypic level there is a positive interaction between music practice and cognitive ability on musical auditory discrimination and achievement [6]. One reason for this could be that general cognitive ability involves cognitive mechanisms that are important for efficient musical practice, e.g., working memory, attention, and sensory discrimination [3]. It remains unknown whether this interaction effect between cognitive ability and practice involves an interaction between genes and the environment (G × E interaction).

Genome-wide association studies (GWASs) offer the opportunity to investigate which genetic variants are associated with a trait of interest. The estimated effect sizes of the genetic variants identified in the GWAS can subsequently be used as weights to calculate a so-called polygenic score (PGS) for an individual. The resulting PGS then is a quantitative measure of an individual's genetic susceptibility for the respective trait studied in the GWAS [14]. Here, we used the PGS approach to investigate whether G × E interaction plays a role in the relations between practice, cognitive ability and expertise in a large cohort of genotyped participants. We used PGSs for (1) cognitive performance (PGScp) and (2) a joint multi-trait measure of cognitive performances, also including educational attainment, highest mathematical school performance and self-rated mathematical ability (PGScps) [15]. After confirming that both PGSs significantly predict cognitive ability, we tested whether PGScp and PGScps are associated with cumulative amount of music practice. This would suggest genetic pleiotropy between music practice and cognitive ability, as shown earlier using other techniques (twin modelling) [9]. Second, we similarly tested for pleiotropy between cognitive ability and musical expertise, by investigating whether PGScp and PGScps are associated with musical expertise outcomes. i.e., lifetime musical achievement and objectively measured musical auditory discrimination [10]. Finally, we investigated whether effects of music practice on musical expertise vary as a function of an individual's PGScp or PGScps (G × E interaction), i.e., if previously reported phenotypic interaction effects [6] involve an interplay between genes and environment.

## 2 Methods

### 2.1 Participants

The Study of Twin Adults: Genes and Environment (STAGE) is a cohort of the Swedish Twin Registry (STR) which includes approximately 32,000 adult twins born between 1959 and 1985 [[16], [17], [18], [19]]. In 2012 and 2013, 11,543 twins from this cohort completed a web survey on, among other things, musical achievement, musical auditory discrimination, music practice and cognitive ability. In 2019 and 2020, individuals from the STR, who provided saliva samples between 2006 and 2008, were genotyped. After quality control, genotype data were available for 8343 individuals from the STAGE cohort, of which 5648 had also completed the web survey in 2012/2013. For analyses with musical auditory discrimination as outcome, the final sample consisted of 3771 individuals (aged between 27 and 54 years old, M = 40, SD = 7.8, of which 1485 males (39.38 %)) for which genotype data, music practice and a musical auditory discrimination score were available; 1564 of these were part of a complete twin pair. For analyses with musical achievement as an outcome, the final sample included 3838 individuals (aged between 27 and 54 years old, M = 40, SD = 7.8, of which 1495 males (38.95 %)), out of which 1560 were part of a complete twin pair. All participants provided informed consent. The study and analyses of biomarkers were approved by the Regional Ethics Review Board in Stockholm, with the approval numbers: Dnr 2011/570-31/5, Dnr 2018/960-31/2, Dnr 2019–05879.

### 2.2 Measures

*Amount of music practice.* Cumulative lifetime music practice of the participants was estimated based on questions about the participant's age when they started and ended (if applicable) playing a musical instrument (including singing) and on the participant's indication of the average intensity of weekly practice during four age intervals (ages 0–5, 6–11, 12–17 and 18 years until time of measurement). Non-players received a cumulative practice score of zero.

*Musical achievement*. Participants filled in an adapted Swedish version of the Creative Achievement Questionnaire (CAQ) [[20], [21], [22], [23]]. This instrument asks participants to rate their lifetime achievement in seven creative domains (visual arts, music, dance, writing, invention, science, and theater) on a seven-point scale. For the music domain this ranges from 1 ‘I am not engaged in music at all’ via 4 ‘I have played or sung, or my music has been played in public concerts in my home town, but I have not been paid for this’ to 7 ‘I am professionally active as a musician and have been reviewed/featured in national or international media and/or have received an award for my musical activities’.

*Musical auditory discrimination* was measured using an online administration of the Swedish Musical Discrimination Test (SMDT) [24]. The SMDT includes three subtests measuring pitch, rhythm, and melody discrimination, in which the participant either indicates whether two presented pitches or rhythms are the same or different, or indicates which tone in the second presented melody was different from the first. The pitch (27 items), rhythm (18 items) and melody (18 items) discrimination scores were standardized before calculating a mean overall musical auditory discrimination score. These kinds of tests have traditionally been used as musical aptitude measures. For additional information on the administration of the SMDT in this sample, see Ullén et al. (2014) [24].

*Cognitive ability.* Cognitive ability or general intelligence was measured with the Wiener Matrizen Test (WMT), a visual matrix test similar to Raven's standard progressive matrices [25]. The test is 25 min long and consists of 24 multiple-choice items, which are scored either one (correct) or zero (incorrect or missing response). A sum of the 24 item scores was used as a measure of the participant's psychometric cognitive ability.

*Level of education.* Participants reported on their level of education obtained, which could range from ‘1' (unfinished primary school) via ‘4' (high school finalized) and ‘7' (bachelor education) to ‘10' (PhD).

*Polygenic scores.* The Polygenic Index Repository offers polygenic scores for a wide range of traits from a number of data sources [15], including the Swedish Twin Registry. The Repository contains single and multi-trait PGSs for 47 phenotypes, among which are single-trait cognitive performance (GWAS sample *N* = 260,354; fluid intelligence, 13 items) and a multi-trait measure based on the same measure of fluid intelligence, educational attainment (years of education and degrees obtained), highest mathematical school performance (most advanced math class successfully completed) and self-rated mathematical ability (GWAS equivalent *N* = 343,411). Multi-trait PGSs use coefficient estimates that are a weighted sum of GWAS coefficient estimates and will, in general, have greater predictive power, but should be interpreted in light of the full set of included traits and their weights, where the weights represent the relative contributions to the predictive power calculated by Multi-Trait Analysis of GWAS (MTAG) [15]. For the multi-trait cognitive performance PGS, the weights were as follows: cognitive performance, 1.37; educational attainment, 0.933; highest mathematical class completed, 1.988; self-rated mathematical ability, 1.045. The Polygenic Index Repository also released 20 principal components (PCs) based on the genome-wide data of each of the participating cohorts, which we merged with our data to control for ancestry structures in the association analyses. More information on the Polygenic Index Repository and details about the PGSs is provided in Becker et al. (2021).

### 2.3 Statistical analyses

All hypotheses were tested using linear regression models in STATA. Sex and age were included as covariates in all analyses; additionally, in all analyses including PGSs, the 20 PCs controlling for ancestry structures were included as covariates. All variables, with the exception of the PCs and sex, were standardized. To correct for relatedness of the twins, we used the robust standard error estimator for clustered observations in STATA [26,27].

We first assessed phenotypic relations between cumulative amount of music practice (including non-players), musical achievement, musical auditory discrimination and cognitive ability. The current sub-sample is part of the larger Humans Making Music study in the STAGE cohort and corresponding phenotypic associations for the full sample have been reported in the past see Mosing et al. (2014, 2019) [6,8]. Next, we tested whether PGScp and PGScps were associated with cognitive ability, music practice, musical achievement and musical auditory discrimination while controlling for sex and age. Lastly, to test our main hypothesis about a G × E interaction, we estimated four regression models in the full sample (including non-players), with music achievement or musical auditory discrimination as the dependent variable and one of the two polygenic scores, i.e., either PGScp or PGScps, as the independent variable, respectively. All of the latter models contained, as independent variables, music practice, the PGS, the PGS × practice interaction as well as interactions between music practice and the 20 ancestry PCs, sex and age [28].

As sensitivity analyses, all analyses were repeated 1) including level of education as a covariate, 2) only in individuals that played music (total N = 2718 for auditory discrimination and N = 2751 for achievement) and 3) excluding one of the twins of a complete monozygotic twin pair (total N = 3268 for auditory discrimination and N = 3318 for achievement).

## 3 Results

### 3.1 Sample descriptives and phenotypic associations

Of the sample, 61 % was female, the mean age at testing was 40 years (SD = 7.8) and 62 % of the participants had obtained a university degree. Both musical achievement and musical auditory discrimination were associated with practice as well as general cognitive ability (Table 1). Notably, achievement showed a stronger association with practice (*β* = 0.67) than with cognitive ability (*β* = 0.17), whereas for auditory discrimination associations with practice (*β* = 0.37) and cognitive ability (*β* = 0.35) had approximately the same magnitude. There was also a weak (*β* = 0.11) association between practice and cognitive ability.

**Table 1:** Results from the different regression analyses separate for each predictor (Model 1–8). Dependent variables are displayed at the top (columns) and independent variables (predictors) on the left (rows). Mean scores and standard deviations for the dependent variables are displayed at the top. Standardized regression coefficients (β) (95 % confidence intervals), *p*-values and explained variance of the respective predictor (ΔR^2^) are displayed.

		Musical achievement			Musical auditory discrimination			Music practice			Cognitive ability		
	Mean (SD)	2.05 (1.42)			13.38 (2.53)			2525.94 (3614.03)			13.36 (5.11)		
Model	**Predictor main effect:**	β	p-value	ΔR^2^	β	p-value	ΔR^2^	β	p-value	ΔR^2^	β	p-value	ΔR^2^
1	Practice	0**.67 (**0**.64–**0**.70)**	**<**0**.001**	0.47	0**.37 (**0**.34–**0**.39)**	**<**0**.001**	0.14						
2	Cognitive ability	0**.17 (**0**.13–**0**.20)**	**<**0**.001**	0.02	0**.35 (**0**.31–**0**.38)**	**<**0**.001**	0.12	0**.11 (**0**.07–**0**.14)**	**<**0**.001**	0.01			
3	PGScp	0**.12 (**0**.08–**0**.16)**	**<** 0**.001**	0.02	0**.16 (**0**.12–**0**.20)**	**<** 0**.001**	0.03	0**.06 (**0**.03–**0**.09)**	**<**0**.001**	0.00	0**.21 (**0**.17–**0**.24)**	**<** 0**.001**	0.04
4	PGScps	0**.14 (**0**.10-**0**.170**	**<** 0**.001**	0.02	0**.16 (**0**.13–**0**.20)**	**<** 0**.001**	0.03	0**.07 (**0**.03–**0**.10)**	**<**0**.001**	0.00	0**.25 (**0**.21–**0**.28)**	**<** 0**.001**	0.06
5	Sex	0**.11 (**0**.05–**0**.16)**	**<**0**.001**	0.00	**−**0**.13 (-**0**.18-.07)**	**<**0**.001**	0.00	0**.09 (**0**.04–**0**.13)**	**<**0**.001**	0.00	−0.24 (−0.31-.18)	<0.001	0.01
6	Age	**−**0**.01 (-**0**.07–**0**.01)**	0**.002**	0.00	**−**0**.09 (-**0**.11-.06)**	**<**0**.001**	0.01	0**.13 (**0**.11–**0**.16)**	**<**0**.001**	0.02	−0.20 (−0.23-.17)	<0.001	0.04
**Interaction effect:**													
7	PGScp × Practice	0**.03 (**0**.00–**0**.06)**	0**.026**		0.02 (0–0.06)	0.179							
8	PGScps × Practice	0**.05 (**0**.02–**0**.08)**	0**.001**		0**.04 (**0**.01–**0**.08)**	0**.010**							

Note: Model 1 and 2 include sex (1 male, 2 female) and age as covariates. Model 3,4, 7 and 8 include sex, age and 20 PCs as covariates. Significant effects and interaction terms are depicted in bold (α = 0.05). Model 7 and 8 testing for interaction effects include main effects.

### 3.2 Genetic pleiotropy

**Fig. 1:**
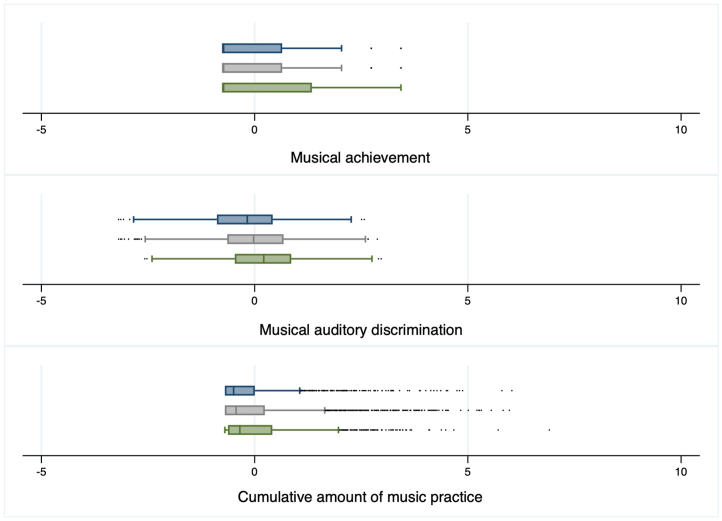
Distributions of the standardized values for musical achievement, musical auditory discrimination and practice for subsamples of participants with polygenic score values below the first quartile (blue: < 25th percentile), the interquartile range (grey: between 25th and 75th percentile) and above the 4th quartile (green: > 75th percentile). Box and whisker plots show the median (vertical line), 25th and 75th percentile, lower and upper adjacent value and outside values for levels of musical achievement, musical auditory discrimination and total amount of music practice. (For interpretation of the references to colour in this figure legend, the reader is referred to the Web version of this article.)

As expected, PGScp and PGScps significantly predicted individuals' general cognitive ability scores (*β* = 0.21- 0.25, *p* < 0.001, Table 1). Furthermore, PGScp and PGScps were significantly associated with musical achievement and musical auditory discrimination (*β* = 0.12 - 0.16, *p* < 0.001, Table 1, Fig. 1). Notably, PGSs were also related to cumulative amount of music practice (*β* = 0.06-0.07, *p* < 0.001, Table 1, Fig. 1), though this effect decreased and became non-significant when controlling for level of education (see Supplementary Table S1). PGScp and PGScps were also related to music practice only in players (*β* = 0.04 and 0.04, *p* = 0.01 and 0.03). Excluding data from one of the MZ twins in each pair did not change the results. Fig. 1 shows distributions for levels of musical achievement, musical auditory discrimination and total amount of music practice for PGS values below the first quartile, the interquartile range and above the 4th quartile.

### 3.3 G × E interaction

**Fig. 2:**
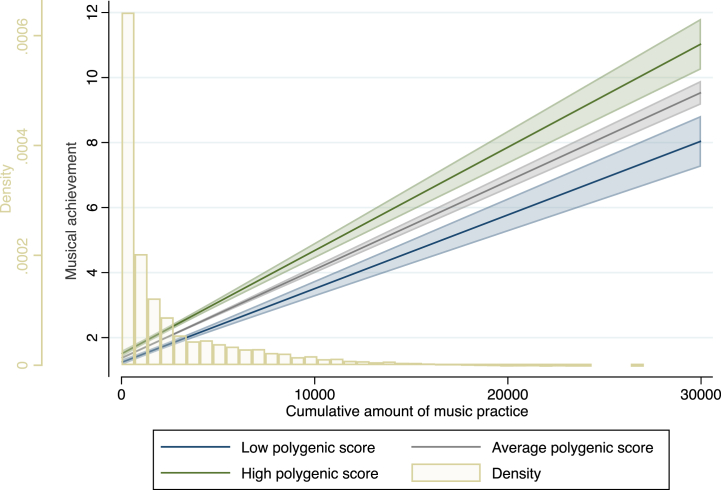
Raw scores for musical achievement and practice hours are shown to ease data interpretation. The linear prediction of cumulative amount of music practice on individuals' levels of musical achievement (95 % CI) with a low (2 standard deviations (SD) below the mean), average (mean) or high (2 SD above the mean) polygenic score for multi-trait cognitive performances, (M = 0, SD = 1, range −3.52, 4.88). Number of participants is reflected in the underlying yellow bar graph. (For interpretation of the references to colour in this figure legend, the reader is referred to the Web version of this article.)

Effects of practice on musical achievement interacted with PGScp and PGScps, while effects of practice on musical auditory discrimination only interacted with PGScps, but not with PGScp (see Table 1). All interaction terms indicated that the association between practice and musical expertise becomes stronger with a higher genetic predisposition for cognitive ability (as an example Fig. 2 shows the interaction between practice and PGScps on musical achievement). Controlling for level of education did not change the results (see Supplementary Table 1), nor did excluding data from one of the twins of each complete MZ twin pair. When repeating the analyses in only musically active participants (players), interactions remained significant with the exception of the music practice × PGScp interaction on music achievement (*β* = 0.03, *p* = 0.09).

## 4 Discussion

Practice is one of the most important and well-studied predictors of expertise. Recent phenotypic studies indicate that practice effects on musical expertise may be moderated by cognitive ability, i.e., that there is a positive practice × cognitive ability interaction. Here, we tested whether this interaction at the phenotypic level may involve interplay between genes and environment, G × E interaction, using PGS based analyses of genotype data. The results supported this hypothesis: when using a PGS for multi-trait cognitive performance, we found small but significant interactions with practice both when using achievement and auditory discrimination as indicators of expertise; for the polygenic score for single-trait cognitive performance, the interaction only reached significance for achievement. Furthermore, we found significant main effects of both PGSs on auditory discrimination, achievement as well as practice, in line with findings from earlier twin studies, demonstrating genetic pleiotropy between cognitive ability, musical auditory discrimination and music practice.

In agreement with past research, we found that the amount of music practice significantly influences musical expertise outcomes, with a much larger effect on lifetime musical achievement than on musical auditory discrimination (R^2^ of 44 % compared to 12 %). This is not surprising as musical auditory discrimination is a narrow ability that may be less amenable to practice effects [24,[29], [30], [31]]. Additionally, we found individuals with a higher genetic predisposition for general cognitive ability to be more likely to practice music, have higher levels of musical achievement, have better musical auditory discrimination and accumulate more music practice hours overall. These findings are also in line with the earlier findings from twin studies that associations between cognitive ability and either musical auditory discrimination or practice partly reflect shared underlying genetic influences (genetic pleiotropy) rather than causal relationships [9,10].

The importance of cognitive ability for educational outcomes in school settings is well-documented. Deary and coworkers, for instance, found a correlation of 0.81 between latent factors representing cognitive ability and educational achievement in a cohort of more than 70,000 English children, with positive associations between cognitive ability and success in all investigated 25 academic subjects [32]. In contrast, the literature on the role of cognitive ability as a moderator of learning effects in domain-specific expertise is sparse. However, Vaci and coworkers, in a longitudinal study of chess players, demonstrated a practice × cognitive ability interaction to the effect that more intelligent players acquired chess skills more rapidly and also reached a higher level of peak performance [33]. Similarly, we have previously demonstrated a positive phenotypic practice × cognitive ability interaction on measures of musical expertise and achievement [6].

Here, for the first time, we could show that genetic differences in cognitive abilities play a role in why individuals might differentially benefit from practice [4,5]. The positive interaction between genetic susceptibility for general cognitive ability and practice on, in particular, musical achievement, indicates that the practice effects on performance increase with cognitive ability. The reasons for this are likely to be complex. One possibility is that higher general cognitive ability is related to more efficient musical skill learning. Cognitive ability is associated with measures of attention [34,35], and it has been shown that tasks that are difficult to automate, when they involve inconsistent novel materials, relate significantly to cognitive ability even after extended periods of practice [4,36]. Clearly, an essential part of practice for musicians is automation of acquired sensorimotor skills through repetition. However, musical experts also need to constantly master new materials and learn new pieces, often in a limited time, and studies have found that working memory capacity predicts sight-reading performance of unfamiliar music, over and above practice [37]. In line with this, Burgoyne and coworkers found that acquisition of piano playing skills in musical novices was significantly predicted by general intelligence, with a β of 0.44 [38]. Notably, large meta-analyses demonstrate that general cognitive ability predicts job performance across a wide range of professions, from unskilled jobs to complex technical and managerial occupations [39]; an important reason for this association appears to be that higher intelligence is related to more efficient acquisition of relevant job knowledge [40].

Further, one should consider that the observed interaction may reflect more general mechanisms, not related specifically to the quality of musical practice. We have previously found music practice to be genetically correlated with a range of music related variables, including openness to experience, intrinsic motivation to engage in music (operationalized as musical flow proneness), and various aspects of musical auditory discrimination [8,41]. Our cumulative measure of music practice could therefore also act as a proxy for musical interest and engagement more broadly. Furthermore, it appears likely that success and real-life achievement in music also depend on non-musical skills, such as social skills and strategical career management, which are associated with cognitive ability. This could be an explanation for why we found a significant interaction for the polygenic score for single-trait cognitive performance only for achievement and not for auditory discrimination. Another explanation for this discrepancy could be the stronger association between practice and achievement compared to practice and auditory discrimination or a lack of power.

Finally, it is important to keep in mind that polygenic scores reflect a mix of both causal genetic signal on the trait under study in the GWAS as well as confounding by environmental contexts like *r*GE. Additionally, the genetic signal can contain confounding due to social, cultural and historical structures (e.g., population structure and geographical locations) [42,43]. This has been shown to be especially true for educational attainment and socio-economic related traits [44,45] and is not unexpected, since a higher socio-economic status offers parents resources to provide their offspring with more nourishing environments (passive *r*GE). Notably, when controlling for education, though interactions remained significant, both the phenotypic and genetic association between practice and cognitive ability diminished, which could indicate that the polygenic score captures some confounding educational attainment effects or that level of education mediates the association between genetic vulnerability for cognitive ability and music practice. A way to disentangle direct genetic effects from PGSs from passive *r*GE and confounding effects like population structure would be to conduct a within-siblings analysis [46]. However, this design relies on complete sibling-pairs and our current sample of 234 complete dizygotic twins would have an insufficient power (23–50%) to detect a significant interaction effect (α = 0.05), taking the correlations between the predictors and outcomes into account [47]. It would be valuable if future studies could further explore the relationship between socio-economic-related traits in relation to cognitive abilities, music practice and musical expertise.

This study has limitations and important caveats to be aware of. As already touched upon, polygenic scores can be confounded by environmental influences. Second, it is important to keep in mind that the use of polygenic scores is limited to individuals of similar ancestries as included in the GWASs (European ancestries). Therefore, findings from this study cannot be generalized across ethnic populations. Third, polygenic scores to-date are based on GWASs that capture only a fraction of the genetic variation underlying behavior traits. Additionally, detecting interaction effects requires larger sample than testing for main effects [48] and replication of findings from G × E research in other samples will therefore be important [49].

To conclude, with the use of polygenic scores, this study shows, that not only phenotypically, but also genetically, cognitive ability, musical expertise and music practice are related. They share underlying genetic influences (genetic pleiotropy). More importantly, genetic differences in general cognitive ability contribute to individual differences in the slope of the relation between practice and expertise. Specifically, effects of practice on musical expertise are somewhat stronger for individuals with a genetic predisposition for higher general cognitive ability, indicating the presence of underlying G × E interaction for the acquisition of skill performance. In other words, differences in expertise are not only to be attributed to practice hours, but genetics also plays an important role in having an easier or more difficult time when acquiring a skill. Overall, results from this study emphasize that when studying factors that influence the development of expertise, such as practice, it is important to also consider gene-environment correlation and interaction and confounding of genetic factors, i.e., genetic pleiotropy.

## Data availability statement

The datasets generated during the current study cannot be made publicly as registry data were used. Individuals are able to apply online at the Swedish Twin Registry to access the twin data.

Our analyses code is available at https://osf.io/h2aus/?view_only=07a7eca06dd24c1793c71e2e2ecd0791.

## CRediT authorship contribution statement

**Laura W. Wesseldijk:** Writing – review & editing, Writing – original draft, Visualization, Methodology, Investigation, Conceptualization. **Miriam A. Mosing:** Writing – review & editing, Writing – original draft, Supervision, Conceptualization. **Fredrik Ullén:** Writing – review & editing, Writing – original draft, Supervision, Investigation, Funding acquisition, Data curation, Conceptualization.

## Declaration of competing interest

The authors declare that they have no known competing financial interests or personal relationships that could have appeared to influence the work reported in this paper.
