# Supplementary material for: Gene-environment interaction in expertise acquisition: Practice effects on musical expertise vary by polygenic scores for cognitive performance
Source: Heliyon. 2024 Jul 6;10(14):e34264. doi: 10.1016/j.heliyon.2024.e34264 (PMC11292230; doi:10.1016/j.heliyon.2024.e34264)
Supplement: Multimedia component 1 [file mmc1.docx]

**Supplementary Table**

**S1**. Controlling for level of education. Results from the different regression analyses separate for each predictor (Model 1 – 7). Dependent variables are displayed at the top (columns) and independent variables (predictors) on the left (rows). Mean scores and standard deviations for the dependent variables are displayed at the top. Standardized regression coefficients (β) (95% confidence intervals), *p*-values and explained variance of the respective predictor (ΔR^2^) are displayed.

|  |  | *Musical achievement* | | | *..* | *Musical auditory discrimination* | | |  | *Music practice* | | |  | *Cognitive ability* | | |
| --- | --- | --- | --- | --- | --- | --- | --- | --- | --- | --- | --- | --- | --- | --- | --- | --- |
| Model | **Predictor main effect:** | β | p-value | ΔR^2^ |  | β | p-value | ΔR^2^ |  | β | p-value | ΔR^2^ |  | β | p-value | ΔR^2^ |
| 1 | Practice | **.66 (.63-.69)** | **<.001** | .44 |  | **.34 (.31-.37)** | **<.001** | .12 |  |  |  |  |  |  |  |  |
| 2 | Cognitive ability | **.12 (.08-.16)** | **<.001** | .02 |  | **.31 (.27-.35)** | **<.001** | .09 |  | **.07 (.03-.10)** | **<.001** | .00 |  |  |  |  |
| 3 | PGScp | **.10 (.06-.13)** | **<.001** | .01 |  | **.13 (.09-.16)** | **<.001** | .02 |  | *.03 (0-.06)* | *.093* | .00 |  | **.17 (.13-20)** | **<.001** | .03 |
| 4 | PGScps | **.10 (.06-.14)** | **<.001** | .01 |  | **.12 (.09-.16)** | **<.001** | .02 |  | *.02 (0-.06)* | *.198* | .00 |  | **.19 (.16-.23)** | **<.001** | .04 |
| 5 | Level of education | **.17 (.15-.20)** | **<.001** | .03 |  | **.22 (.19-.24)** | **<.001** | .04 |  | **.15 (.13-.18)** | **<.001** | .01 |  | **.28 (.24-.31)** | **<.001** | .07 |
|  | **Interaction effect:** |  |  |  |  |  |  |  |  |  |  |  |  |  | |  |
| 6 | PGScp × Practice | **.03 (.01-.07)** | **.020** |  |  | .02 (0-.06) | .129 |  |  |  |  |  |  |  | |  |
| 7 | PGScps × Practice | **.05 (.02-.08)** | **<.001** |  |  | **.04 (.01-.08)** | **.005** |  |  |  |  |  |  |  | |  |

Note: Model 1 and 2 include sex (1 male, 2 female), age and level of education as covariates. Model 3,4, 6 and 7 include sex, age, level of education and 20 PCs as covariates. Significant effects and interaction terms are depicted in bold (α = .05). Model 6 and 7 testing for interaction effects include main effects.
